# Improving Patient Preference Elicitation by Applying Concepts From the Consumer Research Field: Narrative Literature Review

**DOI:** 10.2196/13684

**Published:** 2020-03-31

**Authors:** Niki Ver Donck, Geert Vander Stichele, Isabelle Huys

**Affiliations:** 1 Department of Pharmaceutical and Pharmacological Sciences University of Leuven Leuven Belgium; 2 Health Economics Consultancy ISMS Turnhout Belgium; 3 Digital Health Solution Development MindBytes Merksplas Belgium

**Keywords:** preference elicitation methods, decision making, consumer research methods

## Abstract

**Background:**

Although preference research finds its origins in consumer research, preference elicitation methods have increasingly attracted attention in different decision-making contexts in health care. Simulating real-life decision making is believed to be important during consumer preference elicitation.

**Objective:**

The aims of this study were to compare the process of decision making between patients and consumers and to identify methods from the consumer research field that could be applied in patient preference elicitation.

**Methods:**

A narrative literature review was performed to identify preference elicitation concepts from a consumer context that could offer improvements in health care.

**Results:**

The process of decision making between patients and consumers was highly comparable. The following five concepts from the consumer research field that could effectively simulate a real-life decision-making process for applications in health care were identified: simulating alternatives, self-reflection, feedback-driven exploration, separated (adaptive) dual response, and arranging profiles in blocks.

**Conclusions:**

Owing to similarities in the decision-making process, patients could be considered as a subgroup of consumers, suggesting that preference elicitation concepts from the consumer field may be relevant in health care. Five concepts that help to simulate real-life decision making have the potential to improve patient preference elicitation. However, the extent to which real decision-making contexts can be mimicked in health care remains unknown.

## Introduction

### Background

During the last decade, there has been growing interest in patient perspectives and experiences in health care decision making [1,2]. The idea of patient involvement has become increasingly accepted, as patients are in a unique position to share their day-to-day experiences in dealing with an illness and its treatment. Information about patients’ perceptions and tradeoffs has the potential to inform decision making on different levels. As patients are the end users of medical products, they are the utmost important stakeholder in the context of patient-centered health care and deserve to be involved in medical decision making [3,4]. At the individual level, patients can find themselves in a situation where multiple treatment options exist, without having one option that is clearly superior compared to the others [5]. In some cases, clinical evidence is scarce, resulting in high levels of uncertainty about treatment benefits. In other cases, there is abundant information on the benefits and risks of available options, but patients’ views on the desirability of these outcomes vary greatly, resulting in different opinions of “the best” option [6]. Patients should receive decision support when making these decisions, which are usually referred to as “preference-sensitive decisions” [5,6]. The treating physician can provide decision support to make an informed preference-based choice in the context of shared decision making (SDM) [7]. In this particular context, the process of forming preferences is often referred to as a “value clarification,” which is followed by preference elicitation [6,8]; the combination of these two aspects is called a “value clarification exercise” (VCE) [8]. At the meta level, patient preference data can provide additional information for decisions on drug development, regulatory assessment, or reimbursement [9-13]. Patient preference information is defined by the Food and Drug Administration (FDA) as “Qualitative or quantitative assessments of the relative desirability or acceptability to patients of specified alternatives or choices among outcomes or other attributes that differ among alternative health interventions” [5]. From a societal perspective, the inclusion of the patient opinion could improve the transparency and acceptability of regulatory or reimbursement decisions [2,14]. Finally, the quality of decisions at both the individual and societal levels might increase when decision making is aligned with the patients’ unmet needs [3].

Patient involvement can be realized in a variety of ways: by asking for input from patients via unstructured methods (eg, testimonials, comments in correspondence) or via structured methods (eg, conducting surveys, collecting patient-reported outcomes, or revealing patient preferences) [5,10]. As part of a structured process to reveal preferences, both qualitative and quantitative preference measurement methods can be used.

### Experience of Preference Elicitation in Consumer Research

Quantitative methods for patient preference elicitation include discrete choice experiments (DCEs)/conjoint analysis (CA) or best-worst scaling [11,15]. The DCE technique was introduced by Louviere and Woodworth [16] in the context of marketing to forecast consumer choices. In 1990, CA and DCEs entered the health care setting and have since been increasingly used for patient preference elicitation [17]. Respondents are asked to choose between two or more alternatives, which are usually profiles consisting of different attributes (including product characteristics such as efficacy, adverse events, and mode of administration) and corresponding attribute levels (eg, oral, injection, and inhalation). By analyzing these results, researchers can derive the underlying utility of particular attributes or profiles [17-19]. Despite the application of DCEs in health care for several decades, the resulting data have not yet been systematically applied to societal decision making, and some uncertainties remain about the utility or validity of DCE results in particular decision-making contexts [20,21]. At present, consensus is lacking on how patient preferences can be optimally measured and incorporated into different health care community decision-making processes [2,22].

Since preference research has been conducted for decades in the context of consumers, experiences from this field might further inspire patient preference research [23,24]. Moreover, several innovative approaches to optimize preference elicitation (CA or other techniques) have been explored in the field of consumer research. Indeed, multiple industries offering innovative durable goods rely on preference elicitation methods to guide the development of new products [25]. However, the main difficulty in measuring consumer preferences for new products is the lack of knowledge and experience of respondents with the new product [26]. As these products typically do not yet exist, consumers have no basic understanding about how to assess the importance of new favorable and unfavorable characteristics or how to assess the tradeoffs between these characteristics [26,27]. Examples of such products are personal computers, smartphones, and electric cars [27,28].

Lack of understanding of the basic characteristics of new products resembles a major issue in patient preference elicitation. Considering that almost one in two Europeans have limited health literacy [29], weighing potential risks and benefits could therefore be a very difficult task for laypeople. This poses a challenge, especially in patient preference research, given the association of worse health states with lower levels of health literacy [29]. Furthermore, patients’ medical states might influence their ability to understand the information and engage in a preference elicitation experiment.

### Applying Consumer Research Experience to Inform Health Care Preference Studies

According to Louviere [26], the external validity of DCEs depends on the extent to which all key aspects of a real decision are simulated. Preference elicitation experiments that most closely resemble real choice situations (including framing of situations, relevant contexts, and consequences) should be able to provide real-life results. For this reason, simulations for informational purposes were introduced in consumer research many years ago so that all aspects, ranging from consumer reports, advertising, or even the whole store environment, can be simulated to resemble real-life decision processes as closely as possible [25,28].

Furthermore, when consumers need to construct their preferences while acquiring information, the tradeoffs they consider might be unstable and depend on context effects. Therefore, the results may not reflect true preferences [26,27]. Urban and colleagues offered a method to deal with forecasting problems with new products that they termed “information acceleration” [25,30]. Louviere [26] clearly described the use of information acceleration methodology as follows: “Acceleration of Information Methods rely on multimedia and other technologies to simulate the processes by which individuals become aware of new technologies/products, search for and acquire information about benefits and/or problem solutions, decide whether to consider them and whether they can take advantage of what they offer, decide if they want to buy a product now available, or wait to see how the product market develops and evolves over time.”

In other words, when designing an experiment to elicit patient preferences, patients need to experience the same process as they would in real life. Their lack of knowledge or experience can be overcome by providing the necessary information in a natural way and showing them the results of various options. Simple pictures or videos can be used; however, more interactive simulations allow for more user involvement while better stimulating learning and knowledge retention [31]. Further, Hoeffler [27] stated that consumers who are forced to construct their preferences during an experiment may be unable to provide enduring preferences. The need for deeper consideration of the decision problem is a natural process, which may cause preferences to change over time [6].

The decision process of consumers in the context of a tradeoff situation consists of the following stages: becoming aware of a specific need or a new product, deciding what information to acquire and how to acquire it, deciding which alternatives are available to attain the objectives, forming a utility function or decision rule, and ultimately deciding whether or not to purchase the product (depending on budget or other constraints). Finally, if they decide to purchase, consumers sometimes need to choose which option(s) to purchase [26,32]. Acquiring the right information and learning the different advantages and disadvantages of every option in order to make tradeoffs represents an important step of this process. The situation of naïve consumers might be comparable to that of patients being faced with certain treatment options or a specific disease for the first time. As with consumers, patients need to acquire and process information at a fast pace when confronted with a new product or treatment. CA techniques are well suited to analyze decision making in both cases, as they can either simulate already available alternatives (eg, to compare different therapies available to patients) or elicit preferences for goods that do not yet exist (eg, comparing therapies in the drug pipeline or before market authorization has been obtained). In both cases, using methods that simulate real-life choice situations, such as information acceleration, could potentially be useful in health care. However, a clear comparison between the decision-making process of consumers and patients is lacking, impacting the potential to transfer learnings from consumer to health research situations.

To fill this gap, the aim of this study was to compare the process of decision making between consumers and patients. Furthermore, the goal was to identify consumer research methods or concepts that may improve patient preference elicitation by simulating real-life decisions. Based on this analysis, the applicability of the identified methods or concepts in health care are assessed.

## Methods

### Comparative Description of the Decision-Making Process for Patients and Consumers

The decision-making process of consumers was compared to that of patients. First, the market evolution stages described by Louviere [26] were translated into analog examples for patients engaging in decision making in one of two possible contexts. On the one hand, the context of individual patients engaging in SDM was considered; on the other hand, gathering preference data from a group of patients to inform development, regulatory, or reimbursement decisions was evaluated [7,33].

### Literature Review of Innovative Preference Elicitation Concepts in the Consumer Research Field

A literature search was conducted in the Scopus database to identify innovative concepts from the consumer research field that improve preference elicitation by simulating real-life decisions. Three key terms (Table 1) describing preference elicitation methods that resemble real-life decisions such as DCEs/CA were combined with several terms describing innovation, information methods, and the field of consumer research. Every combination was searched for independently and duplicates were removed during the first step of the process. Papers with a publication date >5 years old (ie, published before 2012) were excluded, as older ideas may have already been applied in the health care context. Finally, only articles in English were included. All identified papers were screened for exclusion based on the title. The exclusion criteria were the following: studies performed in a health care setting (as these papers describe techniques that have already been implemented in health care) and studies without sufficient description of the performed method or describing an actual stated preference experiment. In cases of doubt, papers were retained for a second selection stage. In this second stage, abstracts were reviewed for exclusion based on the above-described and two additional exclusion criteria: describing standard DCEs without any new elements (as described by the current standards for patient preference elicitation) and focusing solely on willingness to pay. The remaining articles were retrieved in full-text form and reviewed in a two-step process by the authors. In the first step, each concept was critically evaluated with respect to its capacity to simulate real-life decisions by one author (NVD). In the second step, another author (GVS) independently reviewed this analysis. Differences were resolved by discussion and, when no consensus could be reached, ties were settled by the third author (IH).

**Table 1:** Search strategy.

Key search term^a^	Combined with (AND)
Preference elicitation	Consumer – Innovative – Scenario based – Simulation – Virtual Reality – Simulation game – Market research – DCE OR conjoint analysis
DCE^b^ or CA^c^	Innovative – Scenario based – Virtual Reality – Simulation game – Market research
measuring preferences OR measure preferences OR preference measurement	Consumer – Innovative – Scenario based – Simulation – Virtual Reality – Simulation game – Market research

^a^The key terms were combined using “AND” with each of the individual terms of column 2 in the same row.

^b^DCE: discrete choice experiment.

^c^CA: conjoint analysis.

### Assessing the Applicability of Innovative Elicitation Concepts for Patient Preference Elicitation

The current standards to conduct CA or DCEs in health care were reviewed based on leading guidelines in the field issued by the International Society for Pharmacoeconomics and Outcomes Research (ISPOR), the US FDA, and the Medical Device Innovation Consortium (MDIC) [5,15,19]. These guidelines served as a baseline to assess the applicability of the identified methods and concepts from the consumer research field in a health care setting. For the applicability assessment, one author (NVD) evaluated each preference elicitation concept against every topic of the three guidelines by defining each concept as relevant or not relevant. The resulting findings were then reviewed by a second author (GVS). In case no consensus could be reached, ties were settled by the third author (IH). For every concept identified, the complementarity to current standards and the rationale for implementation were considered.

## Results

### Comparing the Decision-Making Process for Patients and Consumers

Table 2 presents the health care analogy in both individual and group decision-making contexts, alongside the steps defined in the consumer context [26].

**Table 2:** Different steps of a decision process: health care analogy for the different market evolution stages.

Market evolution stage; Consumer context [26]	Health care analogy	
	Individual context	Group context
Becoming aware of a need Becoming aware of a product	Receiving a diagnosis and becoming aware of (possible) therapies	The experiment is described: patients become aware of different alternatives (therapies)
Deciding what information to acquire and how to acquire it	Deciding what information (on possible treatments) to acquire and how to acquire it, deciding who (eg, family members, caretakers) needs to be involved in the decision-making process	Deciding what information to use that has been made available
Forming decision rules: deciding whether and which options to consider	Forming decision rules: deciding whether and which treatment options to consider	Forming decision rules: deciding whether and which treatment options to consider
Deciding whether to choose now, delay, or never choose	Deciding whether to choose a possible treatment, choose no treatment (eg, watchful waiting), choose to delay treatment, or choose not to be involved in the decision process	Deciding whether to choose a possible treatment or choose no treatment (eg, watchful waiting)
If choosing now, deciding which option to choose	If choosing now, deciding which treatment option (including the option of watchful waiting) to choose	If choosing now, deciding which treatment option (including the option of watchful waiting) to choose

### Concepts from Consumer Research Methods

#### Article Selection and Retrieval

A total of 135 papers were identified using the described search strategy. After selection of titles, 40 papers remained and were screened further by abstract review using the aforementioned criteria. The full text of the resulting 12 papers was analyzed. Five concepts were judged to be potentially interesting for health care and are discussed below. Reasons for excluding the other seven papers were as follows. One paper was excluded as there was no description of a preference experiment, and another paper was judged not to present any innovative ideas, as these turned out to be already included in standard software [34,35]. Further, one paper focused on forecasting decision behavior instead of quantitative preference measurement, and another discussed a compositional approach to evaluate the attributes one by one, which is not complementary with the concept of real-life decision making [36,37]. The concepts of three papers were not applicable to health care: one method could only be applied on very similar products (in the example, different movies were used, whereas the attributes of health care options usually differ greatly); one method presented a framework consisting of 39 engineering parameters that could not be easily translated to health care equivalents; and the last method was particularly useful for products with 70-100 attributes, whereas in health care typically 3-7 attributes are used [38-40]. The five concepts that are potentially interesting in health care are discussed below in turn.

#### Concept 1: Simulating Alternatives

By visualizing alternative land use scenarios, Vignola et al [41] provided a useful method to clarify different options and explore collaboration among stakeholders. The method promotes discussions between stakeholders by presenting the pros and cons of different alternatives and accounting for uncertainties. The scenarios describe possible consequences of different courses of action to improve users' understanding of causal processes associated with every decision. Synthesized images of land use patterns and their consequences on a given landscape are accompanied by a stylized narrative, explaining the key changes depending on the context. Using land development scenarios to represent possibilities in the future has been suggested as a mental exercise to improve planning [42]. Scenario use helps respondents to understand different alternatives and their consequences by improving the cognitive processes in which people collect and combine information and draw inferences [42]. Furthermore, it is recommended to involve all stakeholders as much as possible during the scenario creation phase through interviews, focus groups, and follow-up discussions to refine every aspect [41].

#### Concept 2: Self Reflection

Hauser et al [43] stated that consumers only learn their preferences as they make realistic decisions [43]. To simulate a realistic decision-making process, people need time to self-reflect upon their options. Without self-reflection, preference elicitation methods might not measure enduring (true) preferences, which is in line with Hoeffler’s [27] findings on preferences for new products. In the study, respondents completed three tasks. First, they formed consideration sets of 30 realistic profiles chosen randomly from all available profiles, which means they had to decide whether they would consider buying the product or not for each profile. Next, they performed a structured preference-articulation procedure (Casemap) by selecting the best and worst level per attribute set. The final task was to state their consideration rules in an unstructured email to a friend. One week later, the respondents again formed consideration sets from a random set of 30 profiles. The predictive ability of the articulated preferences was measured with the relative Kullback-Leibler divergence and the predictions were compared with the consideration-set decisions 1 week later. The authors found that self-reflection was facilitated either by completing the 30-profile consideration set or a highly structured Casemap task (as a best-worst exercise). Self-reflection improved a respondent’s capability to articulate preferences that predict consideration sets 1 week later [43]. Finally, the authors suggested that if consumers are asked to articulate their preferences before self-reflection, this articulation would interfere with their abilities to articulate preferences even after they have had a chance to self-reflect [43].

#### Concept 3: Separated (Adaptive) Dual Response

Some preference elicitation methods such as DCEs might encounter problems when “opt-out” options are provided, with respect to both context effects (ie, when a respondent chooses the opt-out option for a reason other than the lack of useful alternative products) and extreme response behavior (ie, respondents will always or never choose the opt-out option under some conditions). Schlereth et al [44] introduced the concepts of separated dual response (SDR) and separated adaptive dual response (SADR) to counter these problems. SDR implies separating forced- and free-choice questions, resulting in the respondents first choosing between two alternatives (forced choice) and then choosing whether or not they actually want the chosen option or would like to opt-out as a second step (free choice). This will overcome the context effects created by dominant alternatives (which decreases the likelihood of selecting the opt-out option) or the existence of very similar alternatives (not choosing is an “easy way out” in this case). SDR also eliminates extreme response behavior since the respondents do not have the opportunity to always or never go for the opt-out option. However, the authors noted that this method might introduce a new context effect of choice deferral, resulting in the respondents more frequently choosing the no-purchase option. They suggested solving this problem by separating the questions in time; that is, asking all forced-choice questions first and all free-choice questions later. SADR contains an extra adaptive mechanism that selects fewer, but more informative, free-choice questions.

#### Concept 4: Feedback-Driven Exploration

Boesch et al [4] proposed the implementation of feedback-driven exploration techniques to improve the validity and reliability when developing a stated-preference experiment. This involves implementing continuous feedback between researchers, respondents, and all other stakeholders throughout the process. The authors formulated the following steps to be included in the research design [45]:

- Shape guiding research questions, concepts, theories, hypotheses.
- Collect and process data.
- Interpret and reflect on data (researcher, possibly with data providers).
- Report tentative research findings to data providers (e.g. survey respondents, interview participants) and broadly review, discuss and explore results with research stakeholders to arrive at overall conclusions,
- Intermediate or preliminary results may indicate a need of getting back to earlier phases of the research process, or even of adjusting and starting the process anew.

The authors suggested that an iterative process (going through the different steps multiple times) might be necessary depending on the research question. Three aspects of a stated-preference experiment are specifically mentioned that may benefit from this approach. First, the validity and reliability of the results can be improved, which is particularly important when dealing with research questions for which no real-life data are available to validate the results. Second, the systematic approach of an overall framework will harmonize all of the different steps required to conduct a preference elicitation experiment. Third, all relevant stakeholders can be involved in the process, whereas experts from outside academia are often overlooked during the research and development phase [45].

#### Concept 5: Arranging Profiles in Blocks to Improve Performance

Adaptive CA consists of two consequential approaches: a composition and a decomposition method. First, respondents evaluate independent attributes (composition method), and then the most preferred attributes are combined in profiles and presented in blocks of two randomly arranged profiles (decomposition method) [46]. This approach is particularly useful when tradeoffs need to be made between a high number of attributes in a user-tailored process. Huertas-Garcia et al [46] suggested a design strategy to improve the performance of the decomposition methodology in adaptive CA by arranging profiles, manually or automated by a computer algorithm, into subsets of two profiles. With this strategy, the respondents are asked to evaluate only a subset of profiles rather than the whole choice set. Dividing the profiles in different blocks has advantages both from behavior and statistical perspectives. Small choice sets are easier to handle and can be assessed faster by respondents. The statistical benefit is that both the variance and covariance of estimations are improved. The aim of this statistical design is to estimate the main factors and two-factor interactions in a quadratic equation with the lowest number of profiles. A limitation of their proposed design is that a maximum of four attributes can be analyzed at the individual level. They argue, however, that this is the average number of preferred attributes obtained after the first step in an adaptive CA.

### Assessing the Applicability of Innovative Elicitation Concepts for Patient Preference Elicitation

#### Current Standards for Patient Preference Studies in Health Care

The ISPOR guideline (as published by Bridges et al [19]) consists of a checklist of 10 topics to be addressed when performing a CA in health care that aims at eliciting preferences at the meta level: Research question, Attributes and levels, Construction of tasks, Experimental design, Preference elicitation, Instrument design, Data-collection plan, Statistical analyses, Results and conclusions, and Study presentation. The MDIC framework focuses on patient preferences regarding benefit-risk assessments of medical device technologies in regulatory decision making [15]. They further provide several topics to consider when developing a preference study, which can be summarized as: defining the research question, the fit of a particular method to the research question, and resources available to undertake a patient preference study. The MDIC guideline discusses both qualitative and quantitative methods and when to use which [15]. The FDA guideline specifically refers to the ISPOR checklist and two other ISPOR guidelines related to good research practices when performing preference elicitation experiments [5,19,47,48]. The major complementarity of the FDA guideline to the other guidelines is its focus on how to inform or educate patients. This is equally important for preference elicitation at the individual or group level.

#### Applicability of Innovative Elicitation Concepts

The five identified concepts provide ideas on how to improve patient preference elicitation. Table 3 displays the assessment of which guideline items could potentially be improved by applying the five identified concepts [5,15,19]. Some concepts are process-oriented and could therefore potentially impact the entire development process. For example, the concept of feedback-driven exploration could have an impact on 9 out of the 10 steps described by the ISPOR guideline [19]. Other concepts focus on specific development steps, or even on more general challenges such as providing information to patients.

**Table 3:** Topics of health care guidelines that might benefit from implementing the identified concepts from the consumer research field.

Guideline and items		Simulating alternatives	Self-reflection	Separated adaptive dual response	Feedback-driven exploration	Arranging profiles in blocks
**ISPOR^a^ guideline**						
	Research question	—^b^	—	—	x^c^	—
	Attributes and levels	—	—	—	x	—
	Construction of tasks	—	x	x	x	x
	Experimental design	—	x	x	x	—
	Preference elicitation	—	x	—	x	—
	Instrument design	x	—	—	x	—
	Data collection	x	—	—	x	—
	Statistical analysis	—	—	—	x	x
	Results and conclusions	—	—	—	x	—
	Study presentation	—	—	—	—	—
Total ISPOR guideline items that could be improved		2	3	2	9	2
**FDA^d^ guideline**						
	Patient centeredness	—	—	—	—	—
	Representativeness of the sample and generalizability of results	—	—	—	—	—
	Capturing heterogeneity of patients' preferences	—	—	—	—	—
	Established good research practices by recognized professional organizations	—	—	—	—	—
	Effective communication of benefit, harm, risk, and uncertainty	x	—	—	—	—
	Minimal cognitive bias	—	—	x	—	—
	Logical soundness	—	—	—	x	—
	Relevance	—	—	—	x	—
	Robustness of analysis of results	—	—	—	—	—
	Study conduct	—	—	—	—	—
	Comprehension by study participants	x	—	—	—	—
Total FDA guideline items that could be improved		2	0	1	2	0
**MDIC^e^ guideline: conjoint analysis and dual response experiments review**						
	Methodology criteria	x	x	x	x	x
	Sample criteria	x	—	—	—	—
	Analysis criteria	—	—	—		x
	Output criteria	—	—	—	x	—
Total MDIC guideline items that could be improved		2	1	1	2	2

^a^ISPOR: International Society for Pharmacoeconomics and Outcomes Research.

^b^—: Guideline topic not impacted by concept implementation.

^c^x: Guideline topic might benefit from concept implementation.

^d^FDA: Food and Drug Administration.

^e^MDIC: Medical Device Innovation Consortium.

## Discussion

### Comparing the Decision-Making Process for Patients and Consumers

The decision-making process of consumers and patients is highly comparable. The main difference lies in the first step of the process, in which patients become aware of the decision context. More cognitive effort might be required to consider all relevant aspects of a health care context relative to that required for a consumer context. The remaining steps of the decision process are the same. For individual patients in the context of SDM, the process is equally comparable, with again only a few small differences. For example, upon receiving a diagnosis of breast cancer, a woman becomes aware of her need for therapy. The treating physician will provide information on the available options such as the possibility of breast-conserving surgery or mastectomy. The patient will then be advised to think about this for a few days and discuss her preferences with friends, family, or fellow sufferers. During a second consultation, the patient’s preferences will be discussed, and a joint decision can be made. In case the patient is not ready to choose or does not want to participate in the decision-making process after all, the physician will propose their preferred option, which is likely to be carried out. The main difference here lies within the step of information gathering. High-quality information on diseases and potential therapy options is usually more difficult to obtain than information on consumption goods. Ideally, the patient receives all of the relevant information, or information sources, from the treating physician. As a second difference, the need for discussing the potential impact of available options with others might be higher in a health care setting than in a consumer setting. The other steps of the decision process are the same for both consumers and patients engaging in SDM. It should be noted, however, that these steps only apply when patients are offered the chance to actively engage in the decision-making process. According to the National Health Service in England, “SDM is relevant in any non-life threatening situation when a health or care decision needs to be made and a range of options (including doing nothing) is available” [49]. Although the process of SDM was introduced in health care decades ago, implementation is still lacking [7,50].

### Applicability of Identified Concepts Within Current Standards for Patient Preference Elicitation

The identified concepts can be useful for one or more aspects of preference elicitation experiments as described by the guidelines. Some concepts can facilitate one or two specific items, whereas others can improve the entire development process. The latter is the case for process-oriented concepts such as feedback-driven exploration. By integrating all of the stakeholders’ opinions in the development process, many aspects of preference experiments could be improved. For example, the attributes and levels could better reflect reality, as there is a smaller chance that relevant items will be left out. The construction of tasks, design, and data collection could be better adapted to patients’ needs, resulting in clearer answers or higher performance rates. The same applies for developing a VCE as part of a decision aid for individual patients. Systematic development guidelines for decision aids already advise to work with a multidisciplinary team including patients and clinicians [51]. All relevant stakeholders should review multiple times and redesign as necessary. Owing to numerous initiatives, patients are now recognized as an important stakeholder in various aspects of health care [1,2,15,22,52]. As the European Patients Forum describes, there has been a transition from “doing things *to* the patient” to “doing things *with* the patient” [53]. Current standards for patient preference elicitation already suggest the use of interviews, and focus groups, among others, to guide the further development in (quantitative aspects of) preference instruments [5,15,19].

The concept of simulating alternatives will mainly improve informational aspects, as it will help people to fully understand the available choice alternatives. This can benefit patients by facilitating the entire process from becoming aware that a decision needs to be made to making that decision. The concept is equally applicable for designing VCEs in an individual context and for developing experiments with a group of patients. Defining the context and effectively communicating the benefits, harms, risks, and uncertainties is one of the first steps in both processes. The importance of this step has been highlighted by the FDA guideline [5]. Properly informing patients has been a longstanding challenge in health care; however, there are no satisfactory guidelines on *how* to do this. The PROTECT Benefit-Risk group compared visual representations to optimally provide information for a benefit-risk assessment [54]. They concluded that multiple formats (ranging from different graphs or plots to pictograms or risk scales) can be considered, and found no single visual type that was superior to others; however, the importance of considering the target audience when choosing a visual format was stressed. The authors further acknowledged the value of interactive/dynamic visuals, which enable active participation and improve understanding [54]. The use of simulating alternatives with photos or video materials seems to be a legitimate and feasible course of action to improve understanding and help create the necessary context to provide information [41]. For instance, researchers could show patients videos of how to use a medication with different modes of administration. This could help them to comprehend precisely what “an injection” entails, or how self-administration compares to administration by a nurse. This could be used by patients who recently underwent surgery and require anticoagulant therapy to prevent thrombosis, as these patients typically can choose between self-administering the injections or having a nurse administer the medication. If patients are shown a video of the complete procedure, including washing one’s hands, disinfecting the skin area, using the right technique to pinch a fold of skin, injecting the syringe at the correct angle, and disposing the needle, they will be better equipped to make the decision of the administration method of the injections. When patients need to make an informed decision, it is important to adequately inform them on the different benefits and risks, but not influence their behavior [55]. That is, we want them to truly understand the benefits and risks, enabling them to make a fact-based decision depending on their values [55].

When all of the relevant information has been provided, respondents need time for self-reflection to let the acquired information sink in and decide which alternative(s) would be the most beneficial in their individual situation [43]. Current standards for patient preference elicitation do not explicitly state requirements concerning time needs to acquire and process information. However, both the FDA and the ISPOR task force warn against information overload or yea-saying, and suggest quizzing the respondents to verify comprehension [5,19]. The MDIC report also expressed the need to gain experience with preference studies and to learn how preferences that change over time can best be evaluated. Implementing this concept by conducting preference elicitation experiments over the course of a few days or weeks might be a good starting point. Researchers could alternatively provide respondents with the necessary information and a preparatory task to think about their preferences on the first day of the experiment. After a few days, the researchers would provide the same information and elicit their preferences during a final preference elicitation task. Of course, the downside to this approach is that the required time per respondent will almost double. Furthermore, the preferences of individuals with chronic diseases might change over time, along will their tradeoffs [15]. In the context of SDM, it is already considered to be good practice to provide patients with a decision aid in preparation for the consultation, as this will allow them to process the information, clarify their preferences, and prepare for a discussion [56].

After exploring the possible alternatives and taking the time to self-reflect, the next step in a decision process is deciding when to choose: now, later, or not at all (see Table 2) [26]. Including an opt-out option (opting not to make a choice or decision) in preference elicitation experiments can simulate the alternative of “choosing not to choose” [44]. In an individual context, this option may translate to watchful waiting or active surveillance. Another possibility is choosing to retain one’s current course of action; for example, when a patient prefers their current treatment over all other options presented. As this situation is realistic, opt-out options should be included in patient preference elicitation experiments whenever relevant. This approach is already supported by the ISPOR health care guideline checklist [19]. An SADR can be used to overcome context effects or extreme response behavior in these cases [44]. However, in cases for which it would not be medically responsible to abstain treatment, this option should not be included in experiments that elicit group preferences [57].

Finally, arranging profiles in blocks of two has the advantage of imposing a low burden on respondents, as it requires less cognitive effort to consider two profiles multiple times rather than multiple profiles a few times [46]. In this way, respondents can repeat the process several times. There is also a statistical advantage, given that with a low number of tasks, doubling the tasks per respondent is equally effective in increasing precision as doubling the number of respondents [58]. The decompositional part of adaptive CA can also be completed with partial profiles, but the main benefit of evaluating two full profiles is that the respondents have the chance to evaluate complete products; this is more similar to the real-life situation by capturing all relevant aspects to consider [26]. Tailoring the choice tasks for the user also fits within a natural decision-making process, as choosing must-have attributes can be a way of forming decision rules. For example, if a preference experiment comprises 10 different attributes, the respondents’ answers could be used to gradually eliminate attributes that are considered less relevant by the respondent, resulting in fewer attributes that are used to form product profiles. This process can only be performed by a computer algorithm, implying the need for a computerized application. As this concept mainly provides statistical benefits, it is less relevant in a context of SDM where only the preferences of an individual patient have to be elicited.

### Differences Between Consumers and Patients: Remaining Challenges

The extent to which we can apply consumer preference elicitation methods to simulate real-life decisions in a health care context is still unclear, both at the individual and meta level. In some respects such as when providing information, these methods could clearly offer improvements to enhance understanding. However, applying consumer preference elicitation concepts in health care will encounter limitations owing to some fundamental differences between health care products and consumables. First, health care is often a very complex matter relative to other consumer needs, making it difficult to fully understand the decision context such as a certain disease or the characteristics of the available options. Trying out different alternatives (eg, different smartphones, cars) is a useful approach in consumer research to obtain information on product characteristics or to determine the option that is most in line with personal needs. However, this solution is simply not possible in health care, as patients cannot test therapy options in the same way that consumers can test a new car. Simulations may be a very helpful alternative, although this will always require a high level of cognitive effort from respondents. Second, the impact of decisions in health care is relatively high, as the decisions are often irreversible. Third, health is an intrinsic part of a person, whereas consumable goods are interchangeable and can be used temporarily. This implies that preference elicitation methods in health care need to provide patients with more complex and more personal information to prepare them for decision making. Fourth, it is important to consider that consumer and health care products are often introduced differently in people’s lives depending on the preference elicitation context. Buying consumables is usually a deliberate decision such as the decision to engage in a preference elicitation experiment for gathering data on market approval or reimbursement. This is different in the individual context, in which the need for health care products can be sudden and unexpected, as is the case upon receiving a diagnosis that is followed by the need to decide on therapy together with the treating physician. Additionally, buying consumables is usually more of an individual decision, whereas the decision-making environment in health care is very complex, often involving multiple stakeholders such as different health care providers, payers, regulatory agencies, and patient advocacy groups. When multiple stakeholder opinions must be taken into account, this impacts the choice of methodology. Finally, another challenge in health care is that preference data can be useful for multiple purposes, ranging from individual to societal decisions. In addition to regulatory authorities, health technology assessment agencies or payers may also take patient preferences into account when making decisions regarding drug approval or reimbursement, respectively. Pharmaceutical companies might be equally interested in using this information to improve drug development. As each stakeholder evaluates preference data from its own perspective, it will be challenging to develop methods that fulfill all needs simultaneously. This versatile use of data is absent in consumer research, where the main goal is to align product development with consumer needs.

### Limitations

The limitations of the study are the following. Only one search engine was used to perform the literature review, and although the list of search terms was quite extensive, it is possible that not all relevant papers were included. The publication date was limited to a maximum of 5 years ago, although older publications might also have concepts that have not yet been introduced in health care. Further, the identified papers were screened for exclusion by only one author, which could have resulted in selection bias.

### Conclusions

The process of decision making is highly comparable between patients and consumers, although some small differences remain depending on the decision-making context. As a result, patients can be categorized as a subgroup of consumers. Therefore, learnings from the consumer research field might be valuable in health care. Five concepts from consumer preference elicitation that could help to simulate real-life decision making were identified in this study. Applying these concepts can result in structural improvements in the development process or improved execution of specific guideline items when eliciting patient preferences. However, the extent to which we can mimic real decision-making contexts in patient preference elicitation requires further research.

## Abbreviations

CA: conjoint analysis
DCE: discrete choice experiment
FDA: Food and Drug Administration
FDE: feedback-driven exploration
ISPOR: International Society for Pharmacoeconomics and Outcomes Research
MDIC: Medical Device Innovation Consortium
SADR: separated adaptive dual response
SDM: shared decision making
SDR: separated dual response
VCE: value clarification exercise

## References

[1] Ho M, Saha A, McCleary KK, Levitan B, Christopher S, Zandlo K, Braithwaite RS, Hauber AB, Medical Device Innovation Consortium’s Patient Centered Benefit-Risk Steering Committee (2016). A Framework for Incorporating Patient Preferences Regarding Benefits and Risks into Regulatory Assessment of Medical Technologies. Value Health.

[2] Mühlbacher AC, Juhnke C, Beyer AR, Garner S (2016). Patient-Focused Benefit-Risk Analysis to Inform Regulatory Decisions: The European Union Perspective. Value Health.

[3] van Til JA, Ijzerman MJ (2014). Why should regulators consider using patient preferences in benefit-risk assessment?. Pharmacoeconomics.

[4] Facey K, Hansen H, Single AV (2017). Patient Involvement In Health Technology Assessment 1st edition.

[5] US Food & Drug Administration (2016). Patient Preference Information – Voluntary Submission, Review in Premarket Approval Applications, Humanitarian Device Exemption Applications, and De Novo Requests, and Inclusion in Decision Summaries and Device Labeling.

[6] Llewellyn-Thomas HA, Crump RT (2013). Decision support for patients: values clarification and preference elicitation. Med Care Res Rev.

[7] Elwyn G, Frosch D, Thomson R, Joseph-Williams N, Lloyd A, Kinnersley P, Cording E, Tomson D, Dodd C, Rollnick S, Edwards A, Barry M (2012). Shared decision making: a model for clinical practice. J Gen Intern Med.

[8] Fagerlin A, Pignone M, Abhyankar P, Col N, Feldman-Stewart D, Gavaruzzi T, Kryworuchko J, Levin CA, Pieterse AH, Reyna V, Stiggelbout A, Scherer LD, Wills C, Witteman HO (2013). Clarifying values: an updated review. BMC Med Inform Decis Mak.

[9] Klein AV, Hardy S, Lim R, Marshall DA (2016). Regulatory Decision Making in Canada-Exploring New Frontiers in Patient Involvement. Value Health.

[10] Danner M, Hummel JM, Volz F, van Manen JG, Wiegard B, Dintsios C, Bastian H, Gerber A, Ijzerman MJ (2011). Integrating patients' views into health technology assessment: Analytic hierarchy process (AHP) as a method to elicit patient preferences. Int J Technol Assess Health Care.

[11] Bewtra M, Johnson FR (2013). Assessing patient preferences for treatment options and process of care in inflammatory bowel disease: a critical review of quantitative data. Patient.

[12] Mühlbacher AC, Zweifel P, Kaczynski A, Johnson FR (2016). Experimental measurement of preferences in health care using best-worst scaling (BWS): theoretical and statistical issues. Health Econ Rev.

[13] European Patients' Academy (2016). Guidance for patient involvement in industry-led medicines R&D.

[14] Bernabe RDLC, van Thiel GJMW, van Delden J (2014). Patient representatives' contributions to the benefit-risk assessment tasks of the European Medicines Agency scientific committees. Br J Clin Pharmacol.

[15] Medical Device Innovation Consortium (2015). Medical Device Innovation Consortium (MDIC) Patient Centered Benefit-Risk Project Report: A Framework for Incorporating Information on Patient Preferences Regarding Benefit and Risk into Regulatory Assessments of New Medical Technology.

[16] Louviere J, Woodworth G (2018). Design and Analysis of Simulated Consumer Choice or Allocation Experiments: An Approach Based on Aggregate Data. J Market Res.

[17] Lancsar E, Louviere J (2008). Conducting discrete choice experiments to inform healthcare decision making: a user's guide. Pharmacoeconomics.

[18] De Bekker-Grob EW, Ryan M, Gerard K (2012). Discrete choice experiments in health economics: a review of the literature. Health Econ.

[19] Bridges JFP, Hauber AB, Marshall D, Lloyd A, Prosser LA, Regier DA, Johnson FR, Mauskopf J (2011). Conjoint analysis applications in health--a checklist: a report of the ISPOR Good Research Practices for Conjoint Analysis Task Force. Value Health.

[20] LaVela SL, Gallan AS (2014). Evaluation and Measurement of Patient Experience. Patient Exp J.

[21] Dirksen CD, Utens CM, Joore MA, van Barneveld TA, Boer B, Dreesens DH, van Laarhoven H, Smit C, Stiggelbout AM, van der Weijden T (2013). Integrating evidence on patient preferences in healthcare policy decisions: protocol of the patient-VIP study. Implement Sci.

[22] Chewning B, Bylund CL, Shah B, Arora NK, Gueguen JA, Makoul G (2012). Patient preferences for shared decisions: a systematic review. Patient Educ Couns.

[23] Johnson FR, Zhou M (2016). Patient Preferences in Regulatory Benefit-Risk Assessments: A US Perspective. Value Health.

[24] Cohen S, Neira L (2003). Maximum Difference Scaling: Improved Measures of Importance and Preference for Segmentation. Sawtooth Software Research Conference Proceedings.

[25] Urban G, Hauser J, Qualls W, Weinberg B, Bohlmann J, Chicos R (2018). Information Acceleration: Validation and Lessons from the Field. J Market Res.

[26] Louviere JJ (2006). What You Don’t Know Might Hurt You: Some Unresolved Issues in the Design and Analysis of Discrete Choice Experiments. Environ Resource Econ.

[27] Hoeffler S (2018). Measuring Preferences for Really New Products. J Market Res.

[28] Urban GL, Weinberg BD, Hauser JR (1996). Premarket Forecasting of Really-New Products. J Marketing.

[29] Sørensen K, Pelikan JM, Röthlin F, Ganahl K, Slonska Z, Doyle G, Fullam J, Kondilis B, Agrafiotis D, Uiters E, Falcon M, Mensing M, Tchamov K, van DBS, Brand H, HLS- E (2015). Health literacy in Europe: comparative results of the European health literacy survey (HLS-EU). Eur J Public Health.

[30] Urban GL, Hauser JR, Roberts JH (1990). Prelaunch Forecasting of New Automobiles. Manage Sci.

[31] Kolb AY, Kolb DA, Armstrong S, Fukami CV (2009). Experiential learning theory: A dynamic, holistic approach to management learning, education and development. The Sage Handbook Of Management Learning, Education And Development.

[32] Louviere J, Henser A, Swait J (2010). Stated Choice Methods Analysis and Applications.

[33] Ali S, Ronaldson S (2012). Ordinal preference elicitation methods in health economics and health services research: using discrete choice experiments and ranking methods. Br Med Bull.

[34] Domigall Y, Albani A, Winter R (2014). Identification of customer preferences for new service development in the electricity domain.

[35] Lancsar E, Swait J (2014). Reconceptualising the external validity of discrete choice experiments. Pharmacoeconomics.

[36] Holm S, Lemm R, Thees O, Hilty LM (2016). Enhancing Agent-Based Models with Discrete Choice Experiments. J Artif Soc Soc Simul.

[37] Eimecke J, Baier D, Lausen B, Krolak-Schwerdt S, Böhmer M (2015). Preference Measurement in Complex Product Development: A Comparison of Two-Staged SEM Approaches. Data Science, Learning by Latent Structures, and Knowledge Discovery.

[38] Rossi S, Barile F, Di Martino S, Improta D (2017). A comparison of two preference elicitation approaches for museum recommendations. Concurr Comp-Pract E.

[39] Wang C (2015). Using the theory of inventive problem solving to brainstorm innovative ideas for assessing varieties of phone-cameras. Comput Ind Eng.

[40] Huang D, Luo L (2016). Consumer Preference Elicitation of Complex Products Using Fuzzy Support Vector Machine Active Learning. Market Sci.

[41] Vignola R, Gonzalez-Rodrigo B, Lane O, Marchamalo M, McDaniels T (2016). A scenario approach to assess stakeholder preferences for ecosystem services in agricultural landscapes of Costa Rica. Reg Environ Change.

[42] Xiang W, Clarke KC (2016). The Use of Scenarios in Land-Use Planning. Environ Plann B Plann Des.

[43] Hauser JR, Dong S, Ding M (2013). Self-Reflection and Articulated Consumer Preferences. J Prod Innov Manag.

[44] Schlereth C, Skiera B (2017). Two New Features in Discrete Choice Experiments to Improve Willingness-to-Pay Estimation That Result in SDR and SADR: Separated (Adaptive) Dual Response. Manage Sci.

[45] Boesch I, Schwaninger M, Weber M, Scholz RW (2012). Enhancing Validity and Reliability Through Feedback-Driven Exploration: A Study in the Context of Conjoint Analysis. Syst Pract Action Res.

[46] Huertas-Garcia R, Gázquez-Abad JC, Forgas-Coll S (2016). A design strategy for improving adaptive conjoint analysis. J Bus Ind Mark.

[47] Hauber AB, González JM, Groothuis-Oudshoorn CGM, Prior T, Marshall DA, Cunningham C, IJzerman MJ, Bridges JFP (2016). Statistical Methods for the Analysis of Discrete Choice Experiments: A Report of the ISPOR Conjoint Analysis Good Research Practices Task Force. Value Health.

[48] Reed Johnson F, Lancsar E, Marshall D, Kilambi V, Mühlbacher A, Regier DA, Bresnahan BW, Kanninen B, Bridges JFP (2013). Constructing experimental designs for discrete-choice experiments: report of the ISPOR Conjoint Analysis Experimental Design Good Research Practices Task Force. Value Health.

[49] NHS England and NHS Improvement (2019). Personalised Care.

[50] Légaré F, Ratté S, Gravel K, Graham ID (2008). Barriers and facilitators to implementing shared decision-making in clinical practice: update of a systematic review of health professionals' perceptions. Patient Educ Couns.

[51] Coulter A, Stilwell D, Kryworuchko J, Mullen PD, Ng CJ, van DWT (2013). A systematic development process for patient decision aids. BMC Med Inform Decis Mak.

[52] European Patients' Academy on Therapeutic Innovation (2017). EUPATI Project: Executive Summary.

[53] European Patients Forum (2015). EPF Background Brief: Patient Empowerment.

[54] Hallgreen CE, Mt-Isa S, Lieftucht A, Phillips LD, Hughes D, Talbot S, Asiimwe A, Downey G, Genov G, Hermann R, Noel R, Peters R, Micaleff A, Tzoulaki I, Ashby D, PROTECT Benefit-Risk group (2016). Literature review of visual representation of the results of benefit-risk assessments of medicinal products. Pharmacoepidemiol Drug Saf.

[55] Fischhoff B, Brewer N, Downs J (2011). Communicating Risks and Benefits: An Evidence-Based User’s Guide.

[56] Stacey D, Légaré F, Col NF, Bennett CL, Barry MJ, Eden KB, Holmes-Rovner M, Llewellyn-Thomas H, Lyddiatt A, Thomson R, Trevena L, Wu JHC (2014). Decision aids for people facing health treatment or screening decisions. Cochrane Database Syst Rev.

[57] Mühlbacher A, Bethge S (2016). First and Foremost Battle the Virus: Eliciting Patient Preferences in Antiviral Therapy for Hepatitis C Using a Discrete Choice Experiment. Value Health.

[58] Johnson R, Orme B (1996). Sawtooth Softw Res Pap Ser.

